# Diagnostic Pitfalls of Discriminating Lymphoma-Associated Effusions

**DOI:** 10.1097/MD.0000000000000800

**Published:** 2015-05-01

**Authors:** Hung-Jen Chen, Kuo-Yang Huang, Guan-Chin Tseng, Li-Hsiou Chen, Li-Yuan Bai, Shinn-Jye Liang, Chih-Yen Tu, Richard W. Light

**Affiliations:** From the Division of Pulmonary and Critical Care Medicine (H-JC, K-YH, S-JL, C-YT); Department of Internal Medicine (H-JC, K-YH, L-YB, S-JL, C-YT); Department of Pathology (G-CT); Division of Hematology and Oncology (L-YB), China Medical University Hospital; Department of Respiratory Therapy (H-JC, S-JL), China Medical University; Department of Internal Medicine (L-HC), Buddhist Tzu-Chi General Hospital, Taichung, Taiwan; and Division of Allergy, Pulmonary and Critical Care Medicine (RWL), Vanderbilt University Medical Center, Nashville, Tennessee, USA.

**Keywords:** CHF, congestive heart failure, CKD, chronic kidney disease, LDH, lactate dehydrogenase, PE, pleural effusion, PMN, polymorphonuclear, PPE, parapneumonic effusion, S-PF, serum minus pleural fluid

## Abstract

High serum lactate dehydrogenase (LDH) level, immunologic defects, enlarged mediastinal lymph nodes, and frequent hydration and diuresis in lymphoma patients may affect the development of pleural effusion (PE). The study was to assess the clinical utility of “Light criteria” and the “recommended algorithm for investigating PEs” in patients with lymphoma.

The characteristics of 126 PEs of lymphoma patients who underwent diagnostic thoracentesis between January 1, 2003, and April 30, 2012, were reviewed. Using Light criteria, 29 (23%) PEs were incorrectly classified. The sensitivity for exudates in Light criteria was 88% and the specificity was only 44%. In 32 transudates, PE LDH correlated with blood LDH concentration (*P* < 0.001, *r* = 0.66). Nine transudates were misclassified as exudates (50%; 9/18) just due to PE LDH more than two-thirds the upper limits. Among the 56 bilateral PEs, 33 (59%) were exudates. Ten (63%) polymorphonuclear (PMN)-predominant exudative PEs were malignant. Infective PEs were often mononuclear (67%) rather than PMN predominant.

When a patient has lymphoma with either unilateral or bilateral PE, thoracentesis for microbiological testing and cytology is imperative. Carefully clinical correlation in addition to the result from Light criteria and differential cell count is essential for prompt management.

## INTRODUCTION

Thoracentesis for the separation of the pleural fluid (PF) into transudates or exudates is the first diagnostic step for patients with undiagnosed pleural effusions (PEs). Transudates occur in patients with increased hydrostatic or decreased osmotic pressure that lead to PE collection. Exudates, however, are the result of direct pleural pathologic changes. If transudates are proven, physicians should treat the underlying cause, such as congestive heart failure (CHF), liver cirrhosis, chronic kidney disease (CKD), or hypoalbuminemia. If the PE is an exudate, cytology or microscopic examination by Gram stain of PE sediment may be helpful.^1,2^

Thoracentesis should not be performed for bilateral PEs in a clinical setting strongly suggestive of a transudate.^1,3,4^ So far, Light criteria have been widely accepted as a means for distinguishing between transudates and exudates based on measurements of lactate dehydrogenase (LDH) and protein in serum and PE.^1,5^

Up to 20%^6^ of patients with non-Hodgkin lymphoma and 30%^7^ of patients with Hodgkin lymphoma reportedly have PE. PEs in lymphomas are usually exudates^8^ and develop by 4 possible mechanisms: by thoracic duct obstruction by a tumor (chylothorax)^9^; by direct pleural involvement of the lymphoma with shedding of cells into the pleural space (malignant PEs)^10^; obstructed lymphatic return due to enlarged hilar or mediastinal lymph nodes (paramalignant PEs)^7,11^; and infections (empyema or parapneumonic effusions [PPEs]).^12^ Nonetheless, in some cases, especially those with advanced stage low-grade lymphomas with multiple organ involvement, the PE may be a transudate.^7^

Most lymphoma patients have high serum LDH levels,^13^ immunologic defects, numerous immature lymphocytes in peripheral blood,^14,15^ and enlarged hilar or mediastinal lymph nodes. The latter results in limited transportation of fluid from the pleural space back to the veins via the lymphatics.^7,11^ Most lymphoma patients also frequently need aggressive hydration and diuresis to avoid tumor lysis syndrome while receiving chemotherapy.^16^

We hypothesized that these characteristics could influence the application of guidelines for the investigation of PEs in lymphoma patients. Bilateral PEs in a lymphoma patient may not be transudates and the percentages of transudative malignant PEs may increase. Furthermore, few lymphoma patients (<3%) were included in previous PE studies of distinguishing between transudates and exudates.^17,18^ The objective of this study was to assess the clinical utility of the “Light criteria”^5^ and the “recommended algorithm for investigation of a PE”^1,3,4^ in lymphoma patients. To our knowledge, the issue has not been reported in the literature except our previous preliminary report.^19^ To date, the present study is 1 of the largest series focusing on PEs in lymphoma patients published.

## MATERIALS AND METHODS

### Identified Patients

There were 774 patients with lymphoma admitted to the China Medical University Hospital, Taichung, Taiwan, an >3000-bed medical center and teaching hospital for referred patients in Taiwan, between January 2003 and April 2012. Lymphoma was confirmed in each patient by either histopathology or immunocytopathology. After approval from the institutional review board (DMR101-IRB2-247), a retrospective analysis was conducted on the medical records of 142 lymphoma patients who had PE in the course of lymphoma treatment and underwent diagnostic thoracentesis. If the patient had bilateral PEs, thoracentesis was done on the predominant side or on PEs with complex-septated sonographic appearances^20,21^ as chest ultrasound is routine practice at our medical center. In cases of PEs requiring repeat thoracentesis, only the first was considered. After excluding 14 patients with incomplete data and 2 patients with transplantation, the study population consisted of 126 patients.

PEs were separated in transudates or exudates after estimation of all patients’ clinical data. All cases were evaluated and classified independently by pulmonologists. Transudates were defined based on imbalances in oncotic and hydrostatic forces, such as CHF, CKD, liver cirrhosis, and hypoalbuminemia with fluid overloading. In contrast, exudates were said to be present when local factors affecting the accumulation of PEs are changed, such as malignant PEs, chylothorax, and PPE.^1,2,4^

The PEs were diagnosed as follows. CHF was diagnosed when depressed ventricular function was confirmed by echocardiography or pulmonary venous congestion, cardiomegaly was present on radiography, and the effusion responded to CHF treatment; liver cirrhosis as the cause of the PEs was diagnosed by clinical evidence and laboratory data of portal hypertension, hepatic injury, and/or ascites; CKD was diagnosed when the glomerular filtration rate was <30 mL/min in the presence of clinical features of fluid overload; hypoalbuminemia was defined by a serum albumin level <30 g/L in the absence of proteinuria and liver cirrhosis; malignant PEs were diagnosed by pleural biopsy specimen, PE cytopathologic study, or significant nodular pleural thickening on computer tomography scans^22^ and response to chemotherapy; chylothorax was diagnosed with a triglyceride concentration of >110 mg/dL in the PE; infection-related PE required an acute disease with pulmonary infiltrates, and responsiveness to antibiotic drug or positive Gram stains or cultures for bacteria in the PEs^18^; and undiagnosed PEs were defined PEs that repeat PE cytology did not demonstrate malignancy and for whom there was no obvious alternative diagnosis.

In all cases of PE caused by CHF, liver cirrhosis, CKD, or hypoalbuminemia with fluid overloading, there was an absence of pulmonary infiltrates associated with an inflammatory process, bacteria isolated from PE, or malignant PE.^18,23^ Moreover, undiagnosed PEs were usually associated with impaired lymphatic return due to enlarged mediastinal or hilar lymph nodes.^7,11^

The routine tests for PE in the study hospital included complete cell count with differential count, biochemical studies of pleural/serum LDH and protein, microbiological testing (ie, Gram stain, aerobic and anaerobic cultures, Ziehl, and mycobacterial culture), triglyceride, and cytology. If the amount of fluid removed exceeded 60 cc, cell blocks and immunohistochemistry were done.^24^ Detailed clinical, radiographic findings, and laboratory results were extracted from medical records.

The Light criteria were tested for differentiating between transudates and exudates in lymphoma patients with PEs. Light criteria included a PF-to-serum protein ratio >0.5, PF-to-serum LDH ratio >0.6, or a pleural LDH concentration >128 IU/L (two-thirds of the upper limit of normal serum LDH concentration [192] at the study hospital). If any 1 of these critical data was met, the PE was an exudate.^5^

“Transudate (L)” was defined as PEs compatible with transudative Light criteria. “Exudate (L)” was defined as PEs compatible with exudative Light criteria. “True transudate” was defined as transudate (L) compatible with a systemic factor (eg, CHF, CKD, liver cirrhosis, or hypoalbuminemia with fluid overloading) induced PE. “False transudate” was defined as transudate (L), but with a local factor (eg, malignancy, chylothorax, or PPE) responsible for the PE. “True exudate” was defined as exudates (L) compatible with a local factor-induced PE. “False exudate” was defined as exudates (L) compatible with a systemic factor-induced PE.

### Statistical Analysis

Continuous variables were reported as mean ± standard deviation whereas categorical variables as numbers (percentage). Linear regression analysis was used to test the relationship between continuous variables. Statistical significance was set at *P* < 0.05. All statistical analyses were performed using the SPSS software, version 17.0 (SPSS Inc., Chicago, IL).

## RESULTS

The characteristics of the 126 lymphoma patients with PEs who underwent diagnostic thoracentesis at the time of presentation were analyzed. There were 92 (73%) B-cell lymphomas, 30 (24%) T-cell lymphomas, and 4 (3%) Hodgkin lymphomas. There were 82 men and 44 women, with mean age of 57 ± 22 years. Fifty-six patients (44%) had bilateral PEs, 36 (29%) had right PEs, and 34 (27%) had left PEs. Their baseline clinical characteristics are summarized in Table 1.

**TABLE 1:**
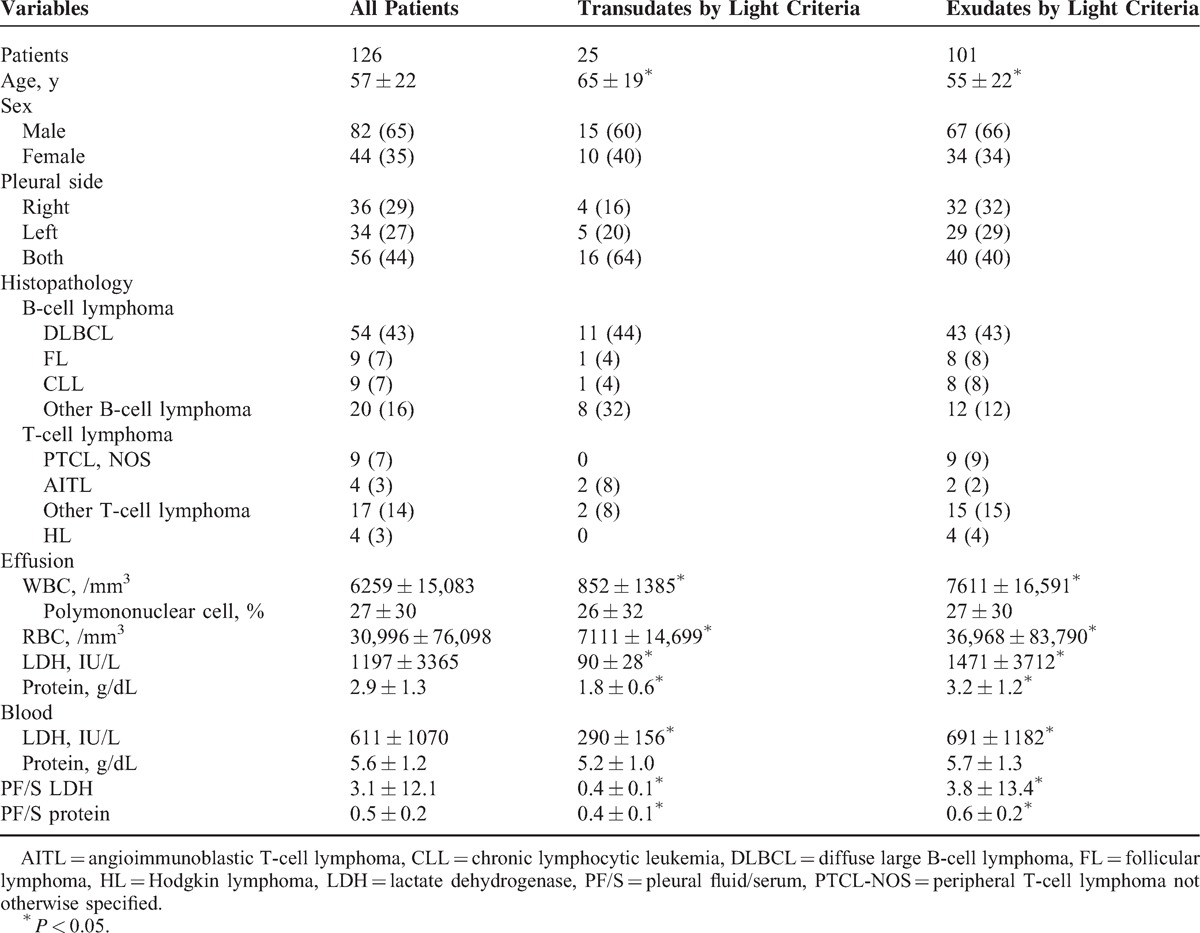
Characteristics of Lymphoma Patients With Pleural Effusion (n = 126) and the Separation of Transudates and Exudates by Light Criteria

Applying Light criteria, 25 (20%) of the PEs were classified as transudates and 101 (80%) as exudates. Variables that differed significantly between the 2 groups by Light criteria were age (transudates 65 ± 19 vs exudates 55 ± 22 years) and blood LDH (transudates 293 ± 156 vs exudates 691 ± 1182 IU/L) (Table 1).

The major etiologies of PEs in lymphoma patients were malignancy (n = 67; 53%), systemic factor-induced transudates (n = 32; 26%), empyema or PPE (n = 19; 15%), pure chylothorax without cancer cells identified or coexisting with empyema (n = 5; 4%), and undiagnosed PEs caused by enlarged hilar or mediastinal lymph nodes with obstructed lymphatic return (n = 3; 2%) (Table 2).

**TABLE 2:**
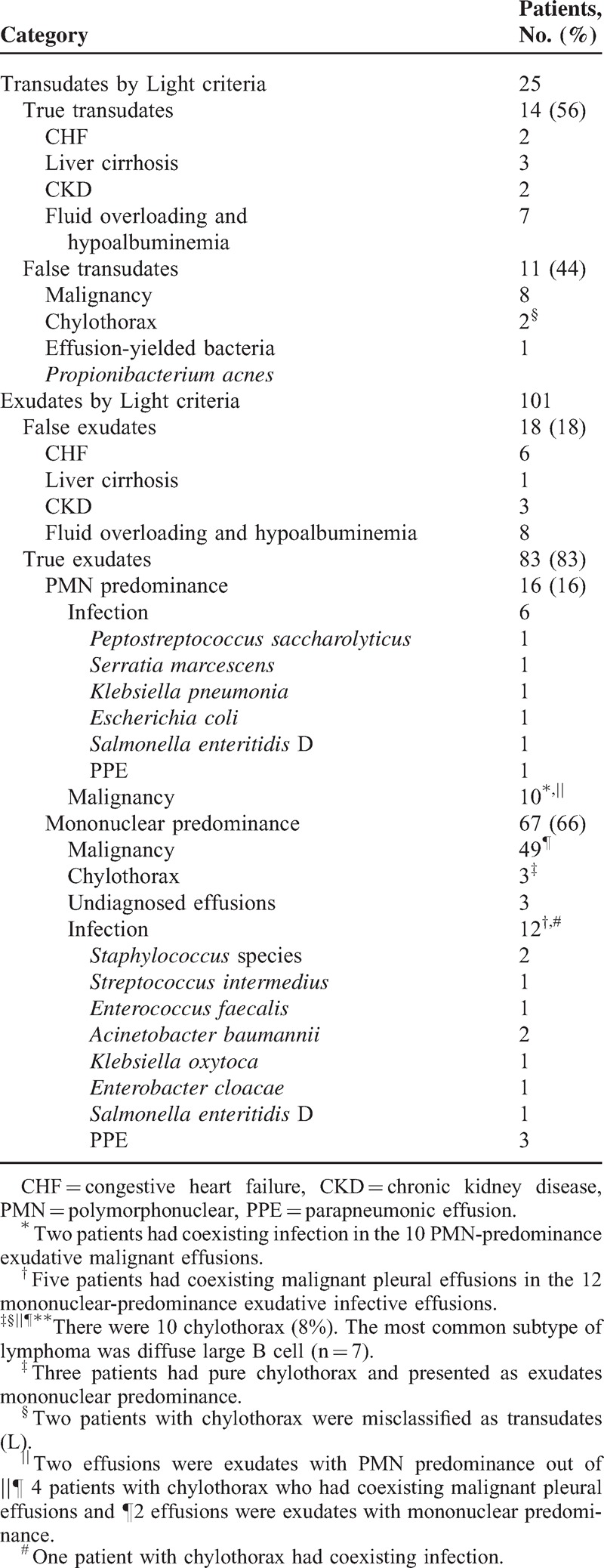
Etiologies of 126 Effusions in Lymphoma Patients and Percentage of Incorrect Classification by Light Criteria

**FIGURE 1:**
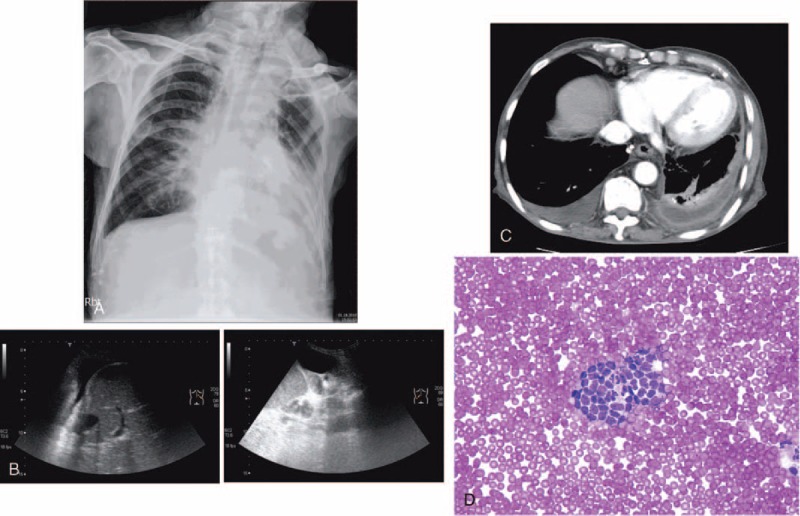
An 83-year-old man had pulmonary marginal zone B-cell lymphoma and bilateral pleural effusions. Effusion analysis via left thoracentesis showed transudates with positive for malignancy. (A) Anteroposterior chest roentgenogram revealed a mass over left retrocardiac area. (B) Ultrasound showed bilateral minimal anechoic pleural effusions. (C) Chest computed tomography revealed significant pleural thickening over left side. (D) Effusion cytology smear showed cluster malignant lymphoid cells. (Liu stain ×400).

Diagnostic accuracy of tests that differentiated exudates from transudates is shown in Table 3. There was no outstanding test for distinguishing between transudates and exudates in 126 lymphoma patients. Using the Light criteria in lymphoma with PEs, 29 (23%) PEs were incorrectly classified. Eighteen transudates were misclassified as exudates (6 secondary to CHF, 1 secondary to liver cirrhosis, 3 secondary to CKD, and 8 secondary to fluid overloading and hypoalbuminemia) and 11 exudates were misclassified as transudates (8 secondary to malignant PEs [Figure 1], 2 due to chylothorax, and 1 due to *Propionibacterium acnes* infection) (Table 2). The sensitivity, specificity, positive likelihood ratio, and negative likelihood ratio for exudate in Light criteria was 88%, 44%, 1.57 and 0.27, respectively (Table 3).

**TABLE 3:**
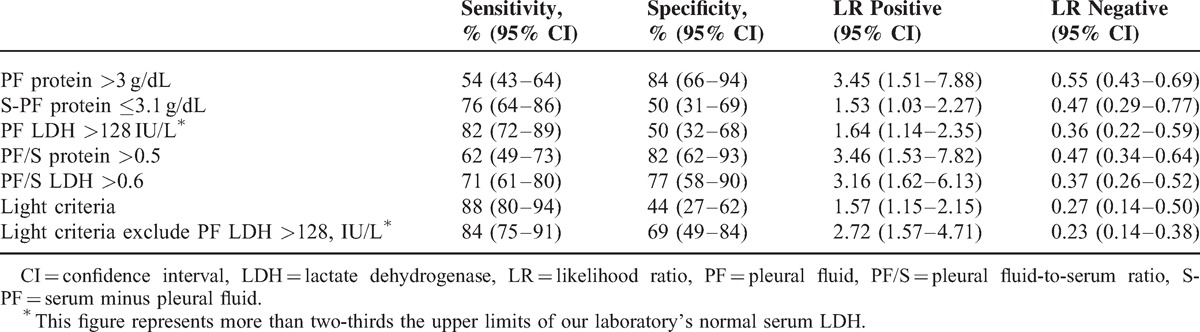
Diagnostic Accuracy of Tests That Identify an Exudative Pleural Effusion in 126 Lymphoma Patients

**FIGURE 2:**
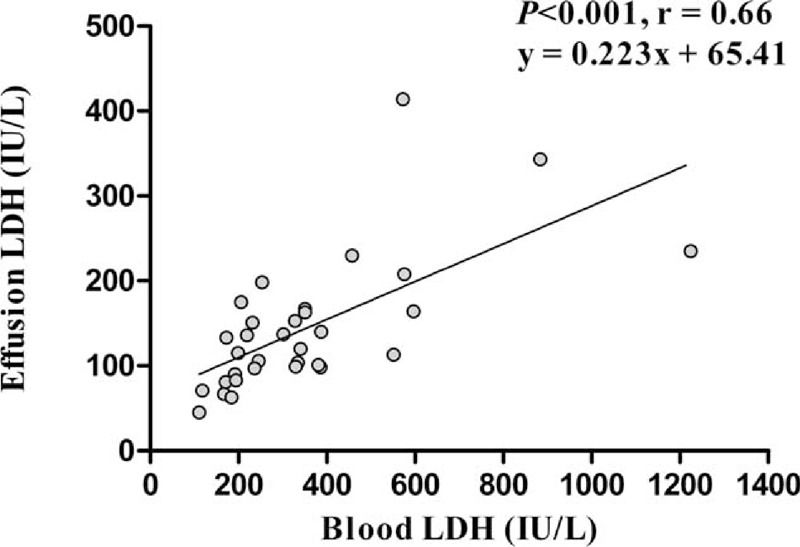
Correlation between effusion and blood levels of LDH in 32 true transudates. LDH = lactate dehydrogenase.

**FIGURE 3:**
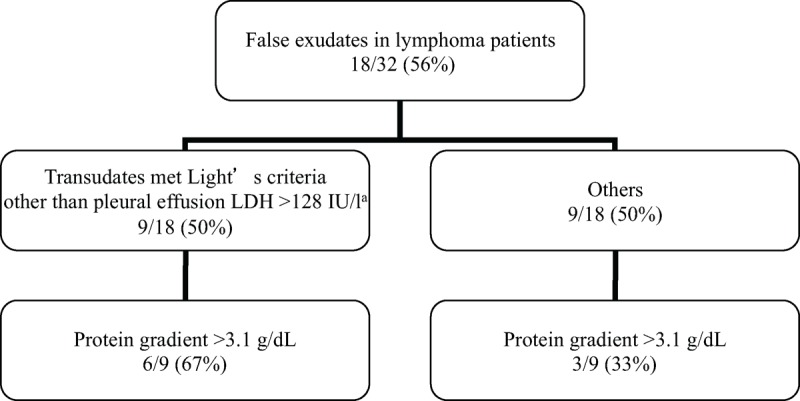
False exudates in lymphoma patients. This flow chart represents the different results of sequential application of protein gradient in lymphoma patients with different false exudates. ^a^This figure represents more than two-thirds the upper limits of our laboratory's normal serum LDH. LDH = lactate dehydrogenase.

Results of linear regression analysis between blood and PEs levels of LDH in 32 transudates (14 true transudates and 18 false exudates) were summarized in Figure 2. The Pearson correlation coefficient (*r*) was significant statistically (*P* < 0.001, *r* = 0.66). Nine transudates were misclassified as exudates (50%; 9/18) due to only a PE LDH >128 IU/L. The specificity and positive likelihood ratio for exudate in Light criteria raised from 44% to 69% and 1.57 to 2.72, respectively, after excluding PF LDH >128 IU/L (Table 3). Serum minus PF (S-PF) protein >3.1 g/dL allowed identification of 67% (6/9) of this category of false exudates. However, for others false exudates, S-PF protein could only identify 33% (3/9) (Figure 3).

**FIGURE 4:**
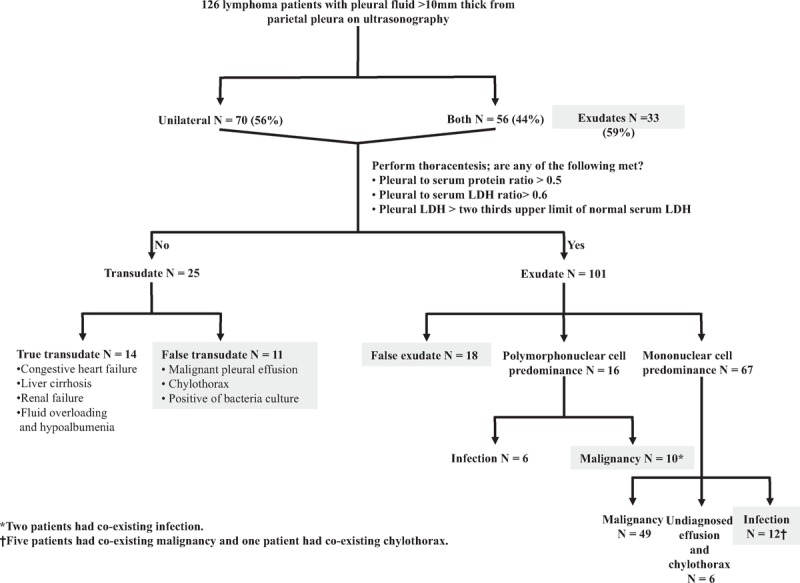
Diagnostic pitfalls of discriminating 126 lymphoma patients-associated effusions. The dark boxes are the conditions that do not follow the general principles of pleural effusion prediction. LDH = lactate dehydrogenase.

**FIGURE 5:**
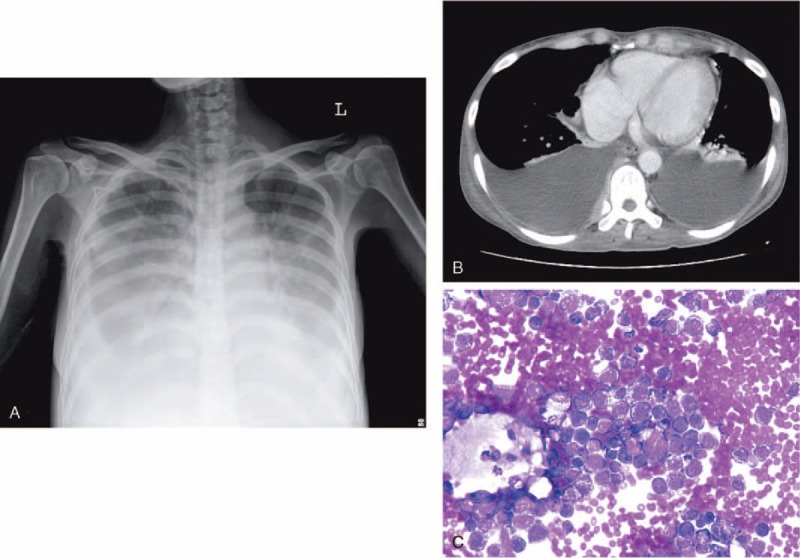
A 34-year-old woman had intestinal diffuse large B-cell lymphoma and bilateral pleural effusions. Effusion analysis via left thoracentesis showed PMN predominance (WBC: 870 /mm^3^ with 56% PMN) and exudative pleural effusion (effusion LDH: 1136 IU/L) positive for malignancy. (A) Anteroposterior chest roentgenogram revealed bilateral ground-glass opacity with meniscus sign. (B) Chest computed tomography revealed prominent bilateral pleural effusions. (C) Cytology smear showed large lymphoid cells with prominent nucleoli. (Liu stain ×400). LDH = lactate dehydrogenase, PMN = polymorphonuclear, WBC = white blood cell.

Among the 56 bilateral PEs, 33 (59%) were exudates (Figures 1, 4 and 5). In true exudates, 16 exhibited polymorphonuclear (PMN) predominance of >50% (range 51%–99%). Five PEs yielded bacteria (including 1 Gram-positive and 4 Gram-negative bacteria) and 1 PPE. The other 10 PMN-predominant exudative PEs were malignant effusions (including 2 patients with superimposed infection) (Figures 4 and 5 and Table 2).

The distribution of 67 mononuclear-predominant exudative PEs included malignancy (n = 49), pure chylothorax (n = 3), undiagnosed PEs (n = 3), and infection (n = 12; 9 with bacteria and 3 PPE). Among the 12 patients with infection, 5 had superimposed malignancy and 1 had superimposed chylothorax (Figure 4 and Table 2).

## DISCUSSION

In this the largest series focusing on PEs in lymphoma patients, the prevalence of PE in lymphoma was 18% (142/774). The major etiology of PEs in lymphoma patients was malignancy. Over half bilateral PEs were exudates. We also showed that using Light criteria in 126 cases of PEs in lymphoma patients, 23% were misclassified. Differential cell count in exudates did not provide diagnostic assistance.

Based on Light criteria, the transudates group was older (transudates 65 ± 19 vs exudates 55 ± 22 years) and the exudates group had higher blood LDH (transudates 293 ± 156 vs exudates 691 ± 1182 IU/L) (Table 1). These findings are compatible with the hypothesis that elderly patients have more CHF and CKD so there is a high possibility of them getting transudates, whereas patients with high serum levels of LDH, an enzyme playing a major role in converting glucose from food into usable energy for cells, suggest an ongoing aggressive disease and will most likely be exudates.

However, Light criteria misclassified 23% (n = 29). The sensitivity for exudates of Light criteria was 88% and specificity was only 44%, unlike previous conclusions of 98% and 83%, respectively.^1^

The high number of false exudates (n = 18) leading to lower specificity of Light criteria in PEs may be due to the repeated chemotherapy and aggressive hydration and diuresis frequently undergone by lymphoma patients.^16^ As previously reported, the weak point of Light criteria is that they sometimes identify a PE in a patient with CHF on diuretics as an exudate.^1,25^ Some lymphoma patients who are receiving chemotherapy may become overhydrated and dehydrated intermittently and this may influence the differentiation of exudates from transudates.

Furthermore, average serum LDH was over 3-fold above the upper limit of normal in the 126 lymphoma patients (LDH 611 ± 1070 IU/L). In 32 transudates (14 true transudates and 18 false exudates), PE LDH correlates with blood LDH concentration (Figure 2). As there is a correlation between the PE LDH and the serum LDH in patients with transudates, a high serum LDH could result in a high PF LDH and the false characterization as an exudate. In this series, in 9 patient only, the PF concentration being greater than two-thirds of the upper limit of normal serum LDH concentration led to 9 transudates erroneously being identified as exudates (50%; 9/18). So, the specificity and positive likelihood ratio for exudate in Light criteria raised from 44% to 69% and 1.57 to 2.72, respectively, after excluding PF LDH >128 IU/L (Table 3). The S-PF protein >3.1 g/dL is a cost-effective test for aiding diagnosis of false exudates.^26^ In 7 studies pooling 857 transudative PEs with a 27.5% of false exudates, 62% would have been correctly labeled by the application of the protein gradients.^27^ In our series, the S-PF protein could only correct 50% (9/18) false exudates. However, for 9 false exudates other than LDH >128 IU/L, S-PF protein >3.1 g/dL allowed correct 67% (6/9), compatible with previous study.^27^ On the other hand, for others false exudates, the correct rate of S-PF protein was only 33% (3/9) (Figure 3).

The PE in patients with positive cytology for malignancy is almost always an exudates (L) and malignant PE is a transudate (L) in only 3.1% to 8%.^28–31^ Cytologic examination should not be performed in all patients with transudates (L) under routine medical practice.^32^ However, in our lymphoma patients, percentages of transudative (L) malignant PEs were as high as 12% (8/67) (Figure 1 and Table 2). Transudates (L) may occur in the early stage of malignant PEs because of lymphoma involvement of the mediastinal lymph nodes, inducing deficient transport of tissue fluids.^31,33^ Furthermore, aggressive hydration in chemotherapy^16^ with presence of coexisting fluid overloading can explain why some malignant effusions are misidentified as transudates.

Chylous PEs resulting from interruption of the thoracic duct or its affluents belong to exudates with mononuclear predominance.^3^ In this study, the rate of chylothorax was 8% (n = 10) and the most common lymphoma subtype was diffuse large B cell (n = 7), compatible with the previous reports.^6,34^ Two patients with chylothorax were misclassified as transudates (L). Two patients with chylothorax coexisting malignant PEs were PMN-predominant exudates (Table 2).

In exudates, if the differential cell count shows a PMN predominance, there is an acute process and the most likely diagnosis is PPE.^1^ Nevertheless, PMN predominance does not rule out the possibility of malignant PEs.^17^ Light et al^17^ identified 18% malignant PEs in 40 PMN-predominant exudative PEs. In the current series of PEs in lymphoma patients, 10 (63%) malignant PEs were confirmed in 16 PMN-predominant exudative PEs (Table 2, Figures 4 and 5).

On the other hand, if the differential cell count shows mononuclear predominance, this indicates a chronic process and cancer or tuberculosis is first considered.^1^ In this series, the major etiology was malignancy (n = 49; 73%) (Table 2). However, 12 infection-induced PEs (4 with Gram-positive bacteria, 5 with Gram-negative bacteria, and 3 PPE) were also identified. It is assumed that lymphoma patients with abnormal lymphocyte reproduction are immunocompromised^14,15^ and there are not adequate PMN cells to fight bacteria.

The current study has several limitations. First, this is a single-center retrospective study, so the generalizability of the results to other hospitals is unknown. Second, serum-PE albumin gradient were not obtained to try and improve accuracy on Light criteria in PEs of lymphoma patients.^25,27^ Third, the rate of chylothorax in PEs of lymphoma patients may be underestimated using the definition of triglyceride concentration of >110 mg/dL in the PE.^35^ Fourth, PE induced by pulmonary embolism may also be underestimated because computer tomography was not performed routinely in lymphoma patients with PEs. Fifth, cases with >1 possible origin of the PE (eg, patients with empyema and malignant PEs) were not excluded. Nevertheless, it may not necessarily influence the final results since only 2 patients had coexisting PPE in 10 PMN-predominant exudative malignant PEs; 5 had coexisting malignant PEs and 1 patient had a coexisting chylothorax in 12 mononuclear-predominance exudative infective PEs.

In conclusion, there are many pitfalls in predicting the nature of PEs of lymphoma patients. Over half bilateral PEs were exudates. Transudates (L) contain malignant cells whereas exudates (L) may be induced by CHF, CKD, liver cirrhosis, and hypoalbuminemia with fluid overloading. The PMN-predominant exudative PEs cannot exclude the diagnosis of malignancy. Infective PEs are often mononuclear rather than PMN predominant. Thus, when a patient with lymphoma (or suspicious lymphoma) presents with either unilateral or bilateral PEs, thoracentesis remains imperative for microbiological testing and cytology. Carefully clinical correlation rather in addition to Light criteria and differential cell count is essential for prompt management.

## ACKNOWLEDGMENT

The authors would like to thank Chia-Ing Li for her technical help in the statistical analysis.
